# Everybody's Page

**Published:** 1891-12-19

**Authors:** 


### Everybody's Page.

"Healthy homes," says one of our contemporaries, "will be in the market when the working man demands them," and in a great measure this is true. The simplest rules of hygiene do not come into the working man's education. Workmen's clubs and institutes please take this to heart! You have many opportunities of teaching elementary rules of health, so why not do something to stimulate the demand and so hasten the supply; it is the business of those who know the value of sanitation to teach him who is ignorant, not to wait till sorrow teaches him the necessity of healthy homes.

---

According to *L'Union Medicale* it will not be long before the removal of tubercular glands by means of an operation will become an antiquated process. It is to be superseded by the injection of the diseased growth with iodoform dissolved in ether, a treatment which is certainly milder, and it is to be hoped more efficient. It is asserted that the injection causes but little pain, and that its effect in causing a disappearance of the disease is rapid. Additional recommendations in favour of the injection are, that it exercises influence at a distance from, as well as at the site, of its introduction, and that no scar is left as is the case after the most careful excision and scraping.

If all that is asserted on behalf of *Cactus grandiflorus* is correct, it promises to prove a very valuable cardiac tonic free from the drawbacks of digitalis. Dr. John Aulde, of Philadelphia, believes that it will effect good results in cases of dilatation, with or without valvular disease, even in cases where digitalis has failed to cause any improvement. He further claims that the alternation of cactus with gelsemium is of great benefit in cases of intermittent heart due to the abuse of tea.

---

The author of a little work entitled, "Health and Hurry," seems to take an unnecessary gloomy view of life. Amongst a large section it is undoubtedly true that dread of being left behind in the race is a motive for more hurry and less rest than are good for health, but there are still plenty of people who are wise and fortunate enough to dispense with the modern exaggerated worship of the goddess of getting on. The regular autumn holiday and the limitation of his working hours are, however, every man's duty, and the modern prevailing fashion of scouting the idea of Sunday as a day of rest, apart from religious principles, is insanitary in the extreme. To the man who wears and tears his very soul out for the sake of filthy lucre life is not worth living, and he is to be pitied deeply by those who, blessed in contentment and moderate "getting on," know how to find the real pleasures of life.

